# *Plasmodium falciparum* proteins involved in cytoadherence of infected erythrocytes to chemokine CX3CL1

**DOI:** 10.1038/srep33786

**Published:** 2016-09-22

**Authors:** Patricia Hermand, Liliane Cicéron, Cédric Pionneau, Catherine Vaquero, Christophe Combadière, Philippe Deterre

**Affiliations:** 1 Sorbonne Universités, UPMC Univ Paris 06, Inserm, Centre d’Immunologie et des Maladies Infectieuses (Cimi-Paris), UMR 1135, ERL CNRS 8255, 91 boulevard de l’Hôpital F-75013, Paris, France; 2 Sorbonne Universités, UPMC Univ Paris 06, Inserm, Centre d’Immunologie et des Maladies Infectieuses (Cimi-Paris), UMR 1135, 91 boulevard de l’Hôpital F-75013, Paris, France; 3 Sorbonne Universités, UPMC Univ Paris 06, Plateforme Post-génomique de la Pitié-Salpêtrière (P3S), UMS 2 Omique, Inserm US029, 91 boulevard de l’Hôpital F-75013 Paris France

## Abstract

Malaria caused by *Plasmodium falciparum* is associated with cytoadherence of infected red blood cells (iRBC) to endothelial cells. Numerous host molecules have been involved in cytoadherence, including the adhesive chemokine CX3CL1. Most of the identified parasite ligands are from the multigenic and hypervariable *Plasmodium falciparum* Erythrocyte Membrane Protein 1 (PfEMP1) family which makes them poor targets for the development of a broadly protective vaccine. Using proteomics, we have identified two 25-kDa parasite proteins with adhesive properties for CX3CL1, called CBP for CX3CL1 Binding Proteins. CBPs are coded by single-copy genes with little polymorphic variation and no homology with other *P. falciparum* gene products. Specific antibodies raised against epitopes from the predicted extracellular domains of each CBP efficiently stain the surface of RBC infected with trophozoites or schizonts, which is a strong indication of CBP expression at the surface of iRBC. These anti-CBP antibodies partially neutralize iRBC adherence to CX3CL1. This adherence is similarly inhibited in the presence of peptides from the CBP extracellular domains, while irrelevant peptides had no such effect. CBP1 and CBP2 are new *P. falciparum* ligands for the human chemokine CX3CL1. The identification of this non-polymorphic *P. falciparum* factors provides a new avenue for innovative vaccination approaches.

As the major causative agent of severe malaria, *Plasmodium falciparum* is responsible for the bulk of malaria-related mortality worldwide^1^. Clinical manifestations of severe malaria result from a combination of high parasite burdens and sequestration of mature *P. falciparum*-infected red blood cells (iRBCs) in microvascular beds throughout the body, a phenomenon described more than a century ago and commonly referred to as cytoadherence^2^. Cytoadherence of iRBC causes obstruction of blood flow in small brain blood vessels - thereby contributing to cerebral malaria - and in the villous chamber of the placenta, leading to pregnancy-associated malaria^3^,^4^,^5^. Sequestration of iRBC causes microvascular obstruction, leads to metabolic disturbances^6^,^7^, and allows iRBC to escape splenic clearance, which is a selective advantage for the microorganism^8^.

Numerous parasite molecules of *P. falciparum* have been identified as ligands for cytoadherence; however, only the surface antigen variant family called “*Plasmodium falciparum* Erythrocyte Membrane Protein 1” (PfEMP1) that is encoded by the *var* genes, is demonstrated as a *bona fide* adherence molecule^9^. Conversely, several host molecules have been identified as receptors for iRBC adherence to human endothelium, including CD36, ICAM1, P-selectin, thrombospondin, CSA (chondroitin sulfate A), and protein C receptor^10^,^11^,^12^. Importantly, clinical data have indicated that cytoadherence of iRBC may involve other molecules that have not yet been identified^13^. For example, iRBC adhere to the chemokine CX3CL1^14^, although the corresponding *P. falciparum* ligand remains unknown.

Chemokines, or chemotactic cytokines, are secreted soluble molecules that are expressed by numerous cell types of the immune system, either constitutively or following the induction of inflammatory responses. Chemokines have indeed played a pivotal role in recruitment, activation, and retention of immune and non-immune cells and specifically, their infiltration or egression at inflamed sites and immune organs^15^. Chemokine receptors are essentially G-protein Coupled Receptors (GPCR), members of the Rhodopsin class^16^. The chemotactic activity of chemokines involves both activation of cell movement as well as adherence on various supports. While chemokines generally operate through cell adherence by inducing or activating classical adhesive molecules like integrins^17^, CX3CL1 is an exception since it is natively expressed as a transmembrane protein that is endowed with an adhesive function unique among chemokines. During inflammation, CX3CL1 is expressed by numerous endothelial cell types, including the cerebral^18^,^19^ and trophoblastic^20^,^21^,^22^ lineages.

Using a proteomic approach, we have identified two *P. falciparum* proteins involved in CX3CL1-mediated cytoadherence. These proteins - named CBP1 and CBP2 for CX3CL1-Binding-Proteins – were previously identified as potential surface proteins of the early gametocytes and called *P. falciparum* Gametocyte-Exported Proteins (GEXP10 and GEXP07)^23^. They have no other known function, share 32% of their residues, and contain a signal peptide and an export motif called “*Plasmodium* export element” (Pexel)^24^ or Vacuolar Transport Signal (VTS)^25^. They have no homology with other proteins from *P. falciparum*, have no ortholog in other *Plasmodium* species and, unlike PfEMP1, PfMC-2TM, RIFIN or STEVOR^26^, are coded by single non–polymorphic genes. CBPs are expressed at the surface of iRBC and mediate the cytoadherence of *P. falciparum*-infected RBC to mammalian cells, a process that can be inhibited by peptides and anti-CBP antibodies.

## Results

### Mature *Plasmodium falciparum*-infected RBC bind specifically to CX3CL1

**Figure 1:**
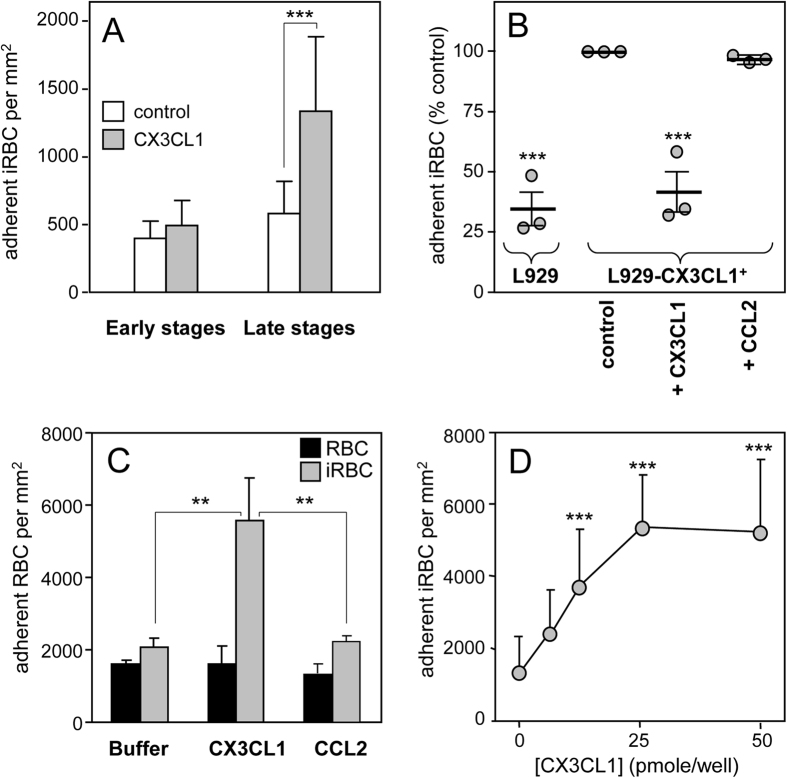
Adherence of 3D7-iRBC on the CX3CL1 chemokine. (**A**) Static adherence of 3D7-iRBC at early stages (12% parasitaemia, 10% ring) or at late stages (7% parasitaemia, 1% ring, 6% mature stages) in 96-well plates coated with 25 pmoles of CX3CL1 (grey bars) or not (control, empty bars). The number of adherent cells per mm^2^ was expressed as mean values and standard deviations from duplicate wells. ***p ≤ 0.0005. Data are representative of three independent experiments. (**B**) Flow adherence of enriched 3D7-iRBC, on parental L929 cells or on CX3CL1 positive-L929 cells after pretreatment or not with 500 nM of CX3CL1 or 500 nM of CCL2. The number of adherent cells was expressed as percent of the control, as mean values and standard deviations from three independent experiments. ANOVA followed by post hoc analysis with Tukey test was performed to establish the levels of significance: ***p ≤ 0.0005. (**C**) Static adherence of normal RBC (black bars) and enriched 3D7-iRBC (grey bars) in 96-well plates coated with 25 pmoles of CX3CL1 or CCL2 per well or not (buffer) as indicated. The number of adherent RBC per mm^2^ was expressed as in A from three independent experiments. ***p ≤ 0.0005. (**D**) Static adherence of enriched 3D7-iRBC in 96-well plates coated with various concentration of CX3CL1. The number of adherent cells per mm^2^ was expressed as in (**A**). The use of enriched iRBC in panels 1C and 1D explains the difference in iRBC adherent number as compared to panel A panel.

RBC infected with mature forms (late trophozoite and schizont) of the 3D7 strain of *P. falciparum* significantly adhered to the immobilized CX3CL1 protein in a static adhesion assay (Fig. 1A) while those infected with parasites at the early stages did not display specific adherence to CX3CL1. We thus decided to work with enriched mature RBC infected with the 3D7 strain, with which all the subsequent experiments were done (quoted 3D7-iRBC). Their adherence was also observed in a flow assay using the L929 fibroblastic cell line that either did or did not express CX3CL1 (Supplementary Figure S1A). The 3D7-iRBCs specifically adhered to the CX3CL1-positive L929 clone, while no adherence was observed on parental cells that did not express CX3CL1 (Fig. 1B). Pre-incubating 3D7-iRBC with soluble CX3CL1 inhibited the adherence, while pre-incubation with the chemokine CCL2 had no effect (Fig. 1B). To further investigate the specificity and selectivity of the 3D7-iRBC adherence, we used a static assay that allows numerous simultaneous conditions. As expected, 3D7-iRBC adhered to immobilized CX3CL1, while non-infected RBC did not (Fig. 1C). Furthermore, this specific adherence was dose-dependent (Fig. 1D) and saturation was reached at 25 pmoles of CX3CL1 per well. Moreover, 3D7-iRBC did not bind to CCL2 (Fig. 1C). Finally, we found that several rounds of selection of 3D7-iRBC on CX3CL1-expressing L929 did not result in the selection of a subpopulation of iRBC with greater adherence to CX3CL1 (Supplementary Figure S1B). So the RBC infected with the 3D7 strain appeared to adhere to CX3CL1 only in their mature form and in a specific and saturable fashion.

### Identification of two CBPs expressed at the surface of iRBC

**Figure 2:**
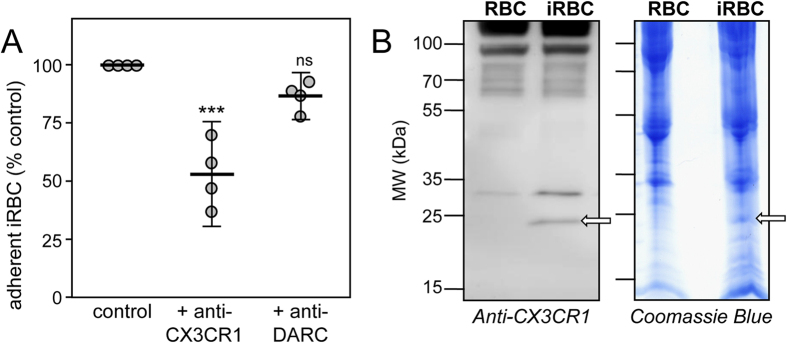
Electrophoresis and anti-CX3CR1 staining of 3D7-iRBC membranes. (**A**) Static adherence to CX3CL1 of enriched 3D7-iRBC pretreated or not (control) with 0.5 μg/ml of anti-CX3CR1 or anti-DARC antibodies, in 96-well plates coated with 25 pmoles. The number of adherent cells was expressed as percent of the control, as mean values and standard deviations from four independent experiments. ANOVA followed by post hoc analysis with Tukey test was performed to establish the levels of significance: ***p ≤ 0.0005; not significant (ns): p > 0.05. (**B**) Membranes proteins of normal RBC and 3D7-iRBC were fractionated on SDS/PAGE and transferred to PVDF membrane, immunostained with anti-CX3CR1 (1/500 dilution, left panel) or stained with Coomassie blue (right panel). Arrows indicates the gel fraction used for mass spectrometry analysis. Arrows indicates the gel fraction used for mass spectrometry analysis. The 20–25 kDa band was the only band specifically appearing in the iRBC membrane lysates (left panel).

As shown by Hatabu *et al*.^14^, 3D7-iRBC adherence is significantly decreased in the presence of an anti-CX3CR1 antibody. In comparison, an antibody raised against DARC (Duffy Antigen Receptor for Chemokines), which is an antigen expressed by both uninfected and infected RBC^27^, had no effect on adherence (Fig. 2A). We therefore hypothesized that the anti-CX3CR1 antibody could recognize a parasitic component expressed on the surface of 3D7-iRBC, which likely shares structural features with CX3CR1. Several proteins were recognized by the anti-CX3CR1 antibody in the extract of 3D7-iRBC membranes; however only one band at 20–25 kDa (Fig. 2B left, arrow) was absent from the membranes of uninfected RBC. Peptides from trypsin-digested extracts of the corresponding 1D gel band from both uninfected and infected RBC (Fig. 2B right, arrow) were analyzed by mass spectrometry (LC-MS/MS) and their sequences were compared to those of proteins encoded by *Plasmodium* genomes in PlasmoDB. Among the hits with significant scores found only in the 1D gel band from iRBC, we selected the corresponding genes coding for proteins with a 20–35 kDa molecular mass and containing a Pexel motif^24^ or VTS (Vacuolar transport signal)^25^, assuming that the potential CX3CL1 ligand should be expressed at the external iRBC membrane (Table 1). These five genes have only one intron but different chromosomal locations. Three of these genes encode for proteins containing a signal peptide and two potential transmembrane domains, similar to most of the Pexel/VTS-containing proteins^26^.

**Figure 3:**
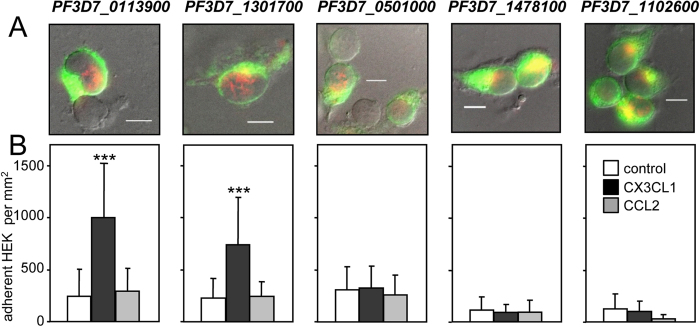
Characterization of HEK293 clones expressing CBP candidates. (**A**) HEK293 clones expressing the five CBP candidates as chimera with EYFP were visualized by transmitted light, wide field EYFP fluorescence (green) and TIRF (red). Merged images indicate the membrane expression of the EYFP-fusion proteins. Bar = 10 μm. (**B**) The clones were subjected to static adherence in 96-well plates coated or not (control) with 25 pmoles of CX3CL1 or CCL2 per well, as indicated. The number of adherent cells per mm^2^ was expressed as mean values and standard deviations from four replicates wells. ANOVA followed by post hoc analysis with Tukey test was performed to establish the levels of significance: ***p ≤ 0.0005.

The five candidate genes were then cloned as fusion proteins with YFP (Yellow fluorescent protein) in HEK293 cells that do not adhere to CX3CL1 or CCL2. The five HEK293 clones expressed the YFP fusion proteins at their cell surface (Fig. 3A), as observed using both wide field fluorescence (Fig. 3A, green) and Total Internal Reflection fluorescence (TIRF) (Fig. 3A, red). Functional adhesion tests revealed that two candidate cell clones, PF3D7_0113900 and PF3D7_1301700, specifically adhered to immobilized CX3CL1 while three others did not (Fig. 3B). For simplicity, PF3D7_0113900 and PF3D7_1301700 are herein referred to CBP1 and CBP2. Adherence of the CBP1- and CPB2-expressing HEK293 clones was dose-dependent (Supplementary Figures S2A and S2C) and specific for CX3CL1 (Supplementary Figures S2B and S2D). In addition to the 3D7 reference strain, transcripts of both *cbp1* and *cbp2* genes were present in samples prepared from the peripheral blood samples of seven patients infected in various African locations (Supplementary Figure S3).

### CBP1 and CBP2 are unique, homologous, monogenic exported proteins with two transmembrane domains, and are expressed on the external surface of iRBC

**Figure 4:**
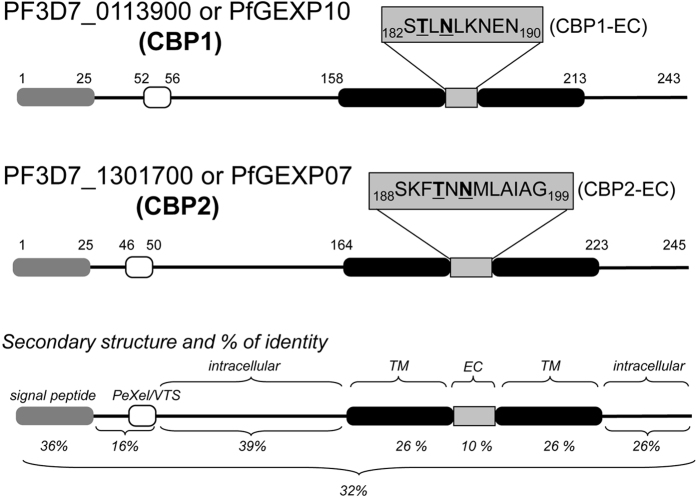
Schematic primary and secondary structure of CBP1 and CBP2. Grey box: signal peptide. Empty box: Pexel/VTS motif. Black box: putative transmembrane domain. The % number indicates the sequence identity between CBP1 and CBP2 domain by domain or as a whole (lower). EC means putative “extracellular domain”.

According to PlasmoDB, CBP1 and CBP2 are monogenic proteins of 243 and 245 amino acids in length, respectively. They are encoded by single-copy genes and their sequences share 32% identity (Table 1, Fig. 4), making them the only two members of the hypothetical family of *Plasmodium* exported protein called “hyp8” (PlasmoDB)^28^. Similar to many genes encoding Pexel-containing proteins, *cbp1* and *cbp2* contain one intron flanked by two exons; there are no homologs in the 3D7 genome and no orthologs in genomes of other *Plasmodium* strains/species, except the chimpanzee *P. reichenowi* one, that is phylogenetically close to the human parasite *P. falciparum*. Both CBP1 and CBP2 have a signal peptide (hence their calculated molecular masses without signal peptide of 25320 Da and 25328 Da, respectively) and two short 9- and 12-amino acid extracellular domains. Their topology may be similar to STEVOR, RIFIN and PfMC-2TM, in that both N- and C-termini are intracellular^26^. Of all the available sequences in the PlasmoDB, the *cbp1* gene has 84 single-nucleotide polymorphisms (SNP) and among them 67 are noncoding or synonymous; *cbp2* has 51 SNP among them 28 are noncoding or synonymous. Remarkably, the non-synonymous SNP of both genes exists outside the regions encoding for extracellular domains.

**Figure 5:**
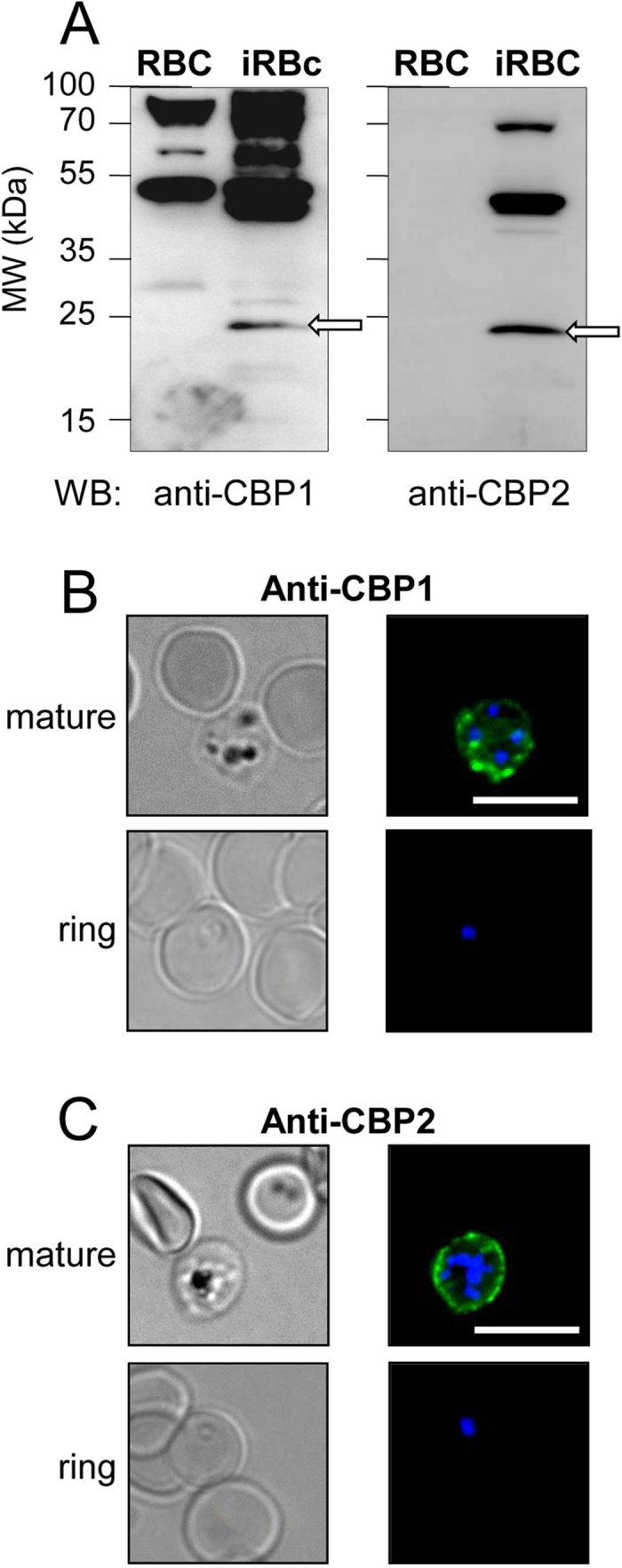
The antibodies raised against CBP1 and CBP2 stain the external membrane of RBC infected by mature 3D7 *Plasmodium falciparum* strain. (**A**) Membranes of untreated RBC and enriched 3D7-iRBC were subjected to Western blotting using anti-CBP1 (left panel) or with anti-CBP2 (right panel) antisera (1/500 dilution). (**B,C**) 3D7-iRBC were visualized by transmitted light, by staining with anti-CBP1 (**B**) or anti-CBP2 (**C**) antisera (green) and by Hoechst staining (blue) using either RBC infected by 3D7 strain at mature stages (80% parasitaemia, after gel flotation) or at early stage (4,5% parasitaemia, 4% ring). Bar = 10 μm.

We next raised rabbit polyclonal antibodies against peptides corresponding to the sequences of the short central domains of CBP1 and CBP2 located between the two transmembrane domains (Fig. 4). In 3D7-iRBC membrane lysates, both anti-CBPs antibodies specifically recognized a 25 kDa band (Fig. 5A, arrows), that matched in size to the band stained by the anti-CX3CR1 serum (Fig. 2 left, arrow). Intact RBC infected with mature - but not immature - forms of 3D7 displayed a peripheral staining either by immunofluorescence (Fig. 5B,C) or weakly by flow cytometry (Supplementary Figure S4). The analysis of immunofluorescence data indicated that this staining occurred in 35 ± 8% (n = 4) and 34 ± 3% (n = 4) of the mature iRBC, for the anti-CBP1 and the anti-CBP2 respectively. Finally, these anti-CBP1 and anti-CBP2 antibodies were used to stain iRBC samples from a patient after a maturation period of 24 hours *in vitro* (Supplementary Figure S5). This showed that CBP1 and CBP2 are expressed at the surface of a significant proportion of the iRBC during natural infection, i.e.33% (22 over 67) for the anti-CBP1 and 23% (22 over 95) for the anti-CBP2. Taken together, these data strongly suggest that CBP1 and CBP2 parasitic proteins are expressed at the external membrane of the iRBC and that the antibodies raised against their potential extracellular domain recognized their respective target.

### Anti-CBP antibodies inhibit iRBC binding to CX3CL1

**Figure 6:**
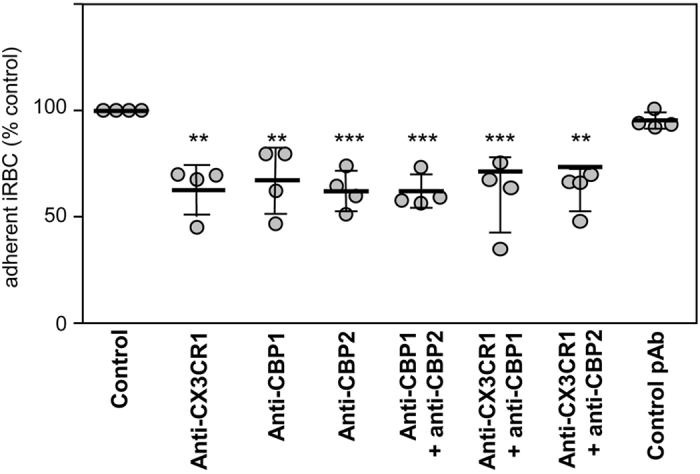
Static adherence of 3D7-iRBC on the CX3CL1 chemokine is decreased in the presence of anti-CBP1/2 antibodies. Static adherence in 96-well plates coated with 25 pmoles of CX3CL1 of enriched 3D7-iRBC pretreated or not (control) with 0.5 μg/ml of anti-CX3CR1 antibodies, affinity-purified anti-CBP1 pAb, affinity-purified anti-CBP2 pAb or addition of both anti-CBP1 and CBP2 pAb, both anti-CX3CR1 and anti-CBP1/2 or with a control pAb. The data are expressed as percent of the control, as mean values and standard deviations from four independent experiments. ANOVA followed by post hoc analysis with Tukey test was performed to establish the levels of significance: **p ≤ 0.005; ***p ≤ 0.0005.

Anti-CBP1 and anti-CBP2 antibodies inhibited the adherence of 3D7-iRBC to immobilized CX3CL1 by 33% and 38% respectively i.e. at a similar level to the anti-CX3CR1 antibody (Fig. 6), while a control rabbit antibody did not have any effect on adherence. When anti-CBP antibodies were incubated simultaneously, the inhibitory effect on adherence was not enhanced. Similarly, incubating the anti-CX3CR1 with either of the anti-CBP antibodies, we did not observe any change in the inhibitory effect (Fig. 6). Along with surface staining (Fig. 5B,C), these observations provided additional evidence to support that CBP1 and CBP2 were both expressed at the surface of iRBC, and that the short central domains were accessible to antibodies and can act as external ligands to host receptors.

### CBP extracellular peptides directly and specifically interact with CX3CL1

**Figure 7:**
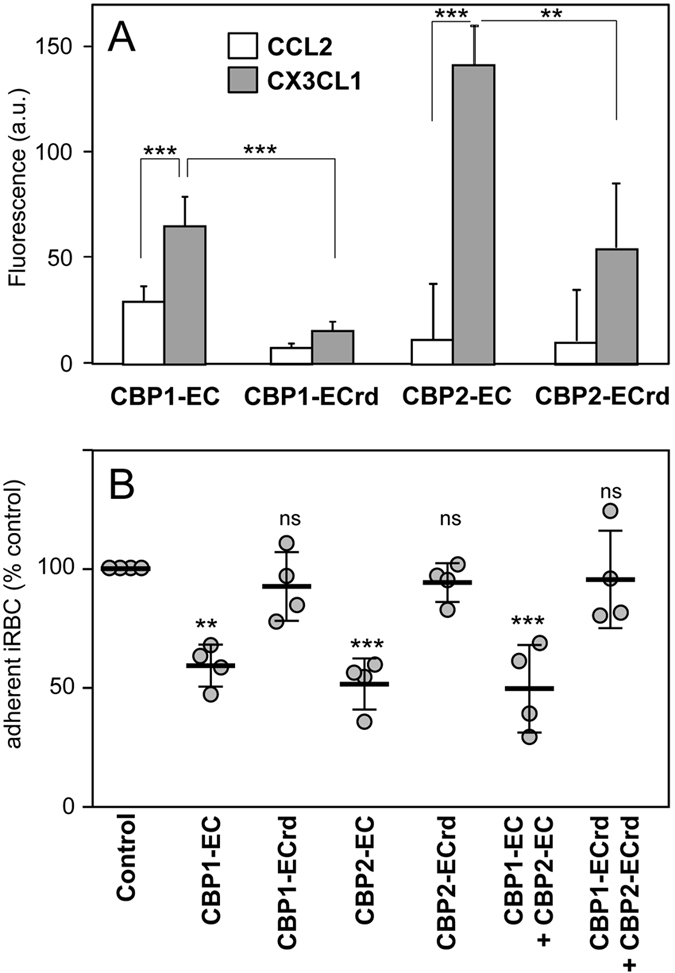
Specific and functional interaction between CX3CL1 and the CBP-EC peptides assayed by fluorescence and static adherence. (**A**) Fluorescence of each of the 5-TAMRA fluorescent peptides were measured in the presence or not of CCL2 or CX3CL1 (excitation 520 nm, emission 580 nm). Experiments are made in triplicate. The background (fluorescence of the control without chemokine) was subtracted and the data were expressed as mean values and standard deviations from three independent experiments. **p ≤ 0.005; ***p ≤ 0.0005. (**B**) Static adherence in 96-well plates coated with 25 pmoles of CX3CL1 of enriched 3D7-iRBC pretreated or not (control) with 0.1 μM of various peptides as indicated. The data are expressed as percent of the control, as mean values and standard deviations from four independent experiments. ANOVA followed by post hoc analysis with Tukey test was performed to establish the levels of significance: **p ≤ 0.005; ***p ≤ 0.0005; not significant (ns): p > 0.05.

We thus hypothesized that peptides corresponding to the predicted extracellular domain (EC) of each CBP (called CBP1-EC and CBP2-EC, Fig. 4) might directly bind to CX3CL1. This assertion was tested using rhodamine-coupled CBP-EC peptides *via* investigating the potential changes of the probe fluorescence in the presence of the chemokine (Fig. 7A). While the fluorescence of CBP1-EC and CBP2-EC only slightly increased in the presence of the control chemokine (CCL2, Fig. 7A, empty bars), it was significantly enhanced in the presence of CX3CL1 (Fig. 7A, grey bars) indicating that the environment of the fluorescent probe was modified by the chemokine binding^29^. By contrast, this fluorescence increase was minimal with the random counterparts of the CBP-EC peptides (CBP1-ECrd, CBP2-ECrd, i.e. peptides with the same residues in a different order) (Fig. 7A). The interaction between CBP and CX3CL1 appeared direct and specific of the chemokine on one hand and of the CBP-EC sequence on the other hand. Moreover CBP1-EC and CBP2-EC peptides significantly inhibited iRBC adherence to CX3CL1 by 41–48% (Fig. 7B), while their random counterparts had no such effect. Lastly the simultaneous addition of both CBP1-EC and CBP2-EC peptides did not enhance this inhibition. Taken together, these data indicate that CBP-EC peptides interact directly and specifically with CX3CL1 and could be used as to functional inhibitors of the iRBC adherence to CX3CL1.

## Discussion

We report here on two parasitic proteins CBP1 and CBP2 that act as *P. falciparum* ligands to CX3CL1, thereby contributing to cytoadherence of iRBC. CBPs are coded by genes of the *P. falciparum* 3D7 reference strain and also in field isolates, as demonstrated by the detection of mRNA transcripts in all patient samples analyzed to date (Supplementary Figure S3). Anti-CBP1 and anti-CBP2 antibodies stained the surface of iRBC from a patient (Supplementary Figure S5) as well as from 3D7-iRBC (Fig. 5B,C, Supplementary Figure S4), suggesting that both CBP1 and CBP2 are expressed at the surface of iRBC.

The intensity of iRBC adherence to immobilized CX3CL1 reached a plateau at 25 pmol of CX3CL1 per well (Fig. 1D). This concentration is very close to that of CD36 and ICAM-1, which supports optimal *in vitro* static adherence of 3D7-iRBC^30^. This CX3CL1-mediated cytoadherence is at least partially mediated by CBP1 and CBP2, since peptides corresponding to their predicted extracellular domains (called CBP1-EC and CBP2-EC) specifically and directly interact with the chemokine (Fig. 7A) and inhibit the binding of iRBC to CX3CL1 (Fig. 7B) as do anti-CBP1 and anti-CBP2 antibodies directed against these short domains (Fig. 6). Similar to the majority of described *P. falciparum* Pexel/VTS-containing genes, the *cbp1* and *cbp2* transcripts are present early in parasite development, while proteins are expressed later at trophozoite and schizont stages, according to PlasmoDB. We showed here that both proteins are expressed at iRBC surface at the late stages (Fig. 5B,C). Our work confirms and extends a previous proteomics approach showing the expression of CBP1 (PF3D7_0113900) at the surface of iRBC^31^. This expression profile was consistent with the iRBC adherence to CX3CL1 primarily observed by Hatabu *et al*.^14^, so it is likely that CPB1 and CBP2 are at least partially responsible of the cytoadherence phenotype observed by this group.

Finally, both CBPs have been reported as exported proteins in early gametocyte proteome^23^, are hence named GEXP10 and GEXP07 (Table 1) and could contribute to the gametocyte sequestration. Actually, the polyclonal antibodies against CPB1 and CBP2 efficiently stained the gametocytes (Supplementary Figure S6). Interestingly, immature gametocytes accumulate in the extravascular spaces of the human bone marrow^32^,^33^,^34^ and it is known that CX3CL1 is well expressed in bone marrow stromal cells^35^, where it contributes to the retention of monocytes^36^. So CX3CL1 could contribute to the gametocyte accumulation in specific body sites, including bone marrow.

The true motif responsible for CBPs binding to CX3CL1 of CBPs remains to be indisputably identified. Interestingly however, cytoadherence-inhibiting-EC domains of CBP1 and CBP2 and the N-terminal peptide of CX3CR1 used to generate antibodies (Fig. 4) share the very short TXN motif (TEN in CX3CR1, TLN in CBP1 and TNN in CBP2). The multiple asparagine residues in the extracellular domain of CBPs are not expected to bind to basic proteins like chemokines. Furthermore simultaneous incubation with both CBP1-EC and CBP2-EC peptides (Fig. 7B) did not lead to any synergistic inhibition (compared to the single-peptide effect) and suggested that both peptides bind to the same site on the CX3CL1 protein. Beyond the context of malaria infection, CBP1-EC and CBP2-EC peptides are very interesting potential hits to initiate the identification of a CX3CL1-specific “neutraligand”. a molecule directly neutralizing the chemokine but not antagonizing the cognate receptor^37^,^38^,^39^.

In analogy with ICAM1 as a host-receptor for *P. falciparum* cytoadherence, CX3CL1 expression is induced on endothelia by inflammation^40^,^41^. This could facilitate the cytoadherence of additional iRBC through CBP-CX3CL1 interaction once the malaria infection has triggered inflammation^10^,^11^,^42^. Whether CBPs act independently from the dominant *P. falciparum* ligand for cytoadherence PfEMP1, or rather in combination with it as a co-ligand, remains to be elucidated.

Although CBP1 and CBP2 induce an iRBC phenotype similar to that induced by PfEMP1 (translation at the ring stage, surface expression and cytoadherence at the mature stage), they differ markedly regarding their structural and genetic features. Our staining (Fig. 5B,C) and functional data (Fig. 6) using antibodies strongly suggest that the N- and the C-terminus of CBP1 and CBP2 are intracytoplasmic (Fig. 4) leaving only short 9- or 12-amino acid domains exposed at the external side of the host red blood cell. In contrast, the PfEMP1 ectodomain is large (250–350 kDa) and built from a combination of multiple domains^43^. By Western blotting (Fig. 5A, left panel), anti-CBP antisera recognized proteins markedly smaller than 200 kDa, thus unequivocally different from PfEMP1. This is consistent with binding assays^44^ ruling out the intervention of PfEMP1 in the adherence phenotype to CX3CL1. Our experiment showing that selection produced no increase in binding (Supplementary Figure S1B) supports the same conclusion. The simplest explanation for the Western blot blotting results with anti-CBP antisera on iRBC membrane lysates (Fig. 5A) is that the 50 kDa and the 75 kDa bands could represent dimer and trimer of the 20–25 kDa CBPs.

The PfEMP1 family is encoded by the highly diverse and polymorphic *var* gene family associated with very broad antigenic diversity. In striking contrast, CBPs are monogenic members of a small family and exhibit no variant in their extracellular domain (PlasmoDB), which is consistent with the fact that we could not select for a subpopulation of iRBC with higher binding affinity even after several cycles of panning (Supplementary Figure S1B). PfEMP1 antigens are highly immunogenic; however intra-clonal diversity and poorly overlapping antigenic repertoires between clones/strains is a major drawback in vaccine development. Conversely, CBPs are constitutively expressed and monogenic and, as such, expected to be poorly immunogenic. This hypothesis was confirmed by our observation that all the plasma from patients living in (or recent migrants from less than 2 years) high malaria endemic countries (West and Center Africa) were found to be negative in an ELISA assay against the CBP1-EC and CBP2-EC peptides (n = 19, data not shown, Supplementary Method). If this poor immunogenicity of CBPs can be circumvented by an innovative vaccine design, the final outcome may have a major impact on vaccine-induced control of *P. falciparum* malaria.

In conclusion, the identification of parasite ligands for the endothelial CX3CL1 offers new ways to prevent malaria, and provides a novel, non-polymorphic target on iRBC that could be used for vaccination, a long-awaited weapon in times of increasing parasite resistance to antimalarial agents^45^,^46^.

## Methods

### Materials

All chemicals are from Sigma-Aldrich (L’Isle d’Abeau, France) except when stated. Chemokine CCL2 was from Peprotech (Levallois-Perret, France) and chemokine domain of CX3CL1 from R&D Systems (Lille, France). The peptides CBP1-EC (STLNLKNEN) and CBP2-EC (SKFTNNMLAIAG) as well as the random peptides CBP1-ECrd (NLETNSKNL) and CBP2-ECrd (MATGSAKNLFNI) were synthetized in the Institut de Biologie Paris Seine (UPMC). Anti-CX3CR1 polyclonal antibody (pAb 14-6093) was purchased from eBioscience (SAS, Paris, France). Anti-DARC antibody was a generous gift from O. Bertrand (Inserm U-1134). The anti-CBP1 and anti-CBP2 polyclonal antibodies were performed at Proteogenix (Schiltigheim, France) by immunizing rabbit with two synthetic peptides, LSTLNLKNEN and AATSKFTNNMLAIAGVG respectively. The 5-TAMRA fluorescent CBP-EC peptides were obtained by adding a L-5-Carboxytetramethylrhodamine at the C-terminal and were synthetized by Proteogenix (Schiltigheim, France). Human embryonic kidney (HEK293) and the fibroblastic L929 cell lines were grown in DMEM medium supplemented with 10% fetal calf serum (Dutscher, Brumath, France), 1% sodium pyruvate and antibiotics. To obtain L929 clone expressing CX3CL1, the CX3CL1-pEYFP plasmid^47^ was transfected into L929 cells with the cationic polymer transfection reagent JetPEI (Polyplus transfection, Ozyme, St Quentin-en-Yvelines, France). A stable transformant resistant to 0.5 mg/ml G418 (Invitrogen) was selected with a FACS Aria cell sorter (BD Biosciences, Le Pont de Claix, France). The *Plasmodium falciparum* 3D7 cloned strain was cultured in human erythrocytes as described previously^48^. Enrichment of late-stage infected erythrocytes (parasitaemia greater than 80%) was obtained by gelatin flotation (Plasmion, Fresenius Kabi, France) according to already published protocol^49^.

### Ethics statement

Clinical isolates were derived from patients’ blood samples obtained for diagnosis purposes and collected under patient approval (written informed consent) by the CNR team (Centre National de Référence du Paludisme pour la France Métropolitaine) according to a procedure common to all National Reference Centers (CNR; http://www.invs.sante.fr/Espace-professionnels/Centres-nationaux-de-reference/Textes-reglementaires) in France. Use of clinical samples and experimental protocols were done in accordance with the relevant guidelines and were approved by the Ile-de-France VI Institutional Review Board.

### Identification of CX3CL1-Binding Protein (CBP) candidates by SDS-PAGE and mass spectrometry

Membranes from RBCs and iRBCs (parasitaemia greater than 80%) were prepared by hypotonic lysis^50^ by incubation in 5P8 buffer (5 mM NaH_2_PO_4_/Na_2_HPO_4_, 0.35 mM EDTA, pH 8) and centrifuged 5 min at 4000 × g at 4 °C to remove parasite pellet. The supernatant containing RBC ghost was centrifuged 15 min at 25,000 × g at 4 °C. Then the pellet was washed in 5P8 buffer by centrifugation several times until the ghost pellet became white. Ghosts were lysed in PBS buffer containing 1% Triton-X100, 0.5% SDS and anti-protease cocktail Complete (Roche Diagnostics, Meylan France). After centrifugation for 45 min at 25,000 × g (4 °C), protein concentration of supernatants was evaluated (BCA, Pierce, Thermo Fisher Scientific, Courtaboeuf, France). Proteins were separated by 4–20% SDS-PAGE (Novex 4–20%, Life Technologies). The gel-separated proteins were either stained with Coomassie Blue (120 μg of cell lysate per lane) or transferred to PVDF membranes (Dutscher) (50 μg of cell lysate per lane). Immune complexes were visualized with secondary peroxidase-conjugated antibodies using a chemiluminescent kit (GE Healthcare Europe, Saclay, France). For protein identification by mass spectrometry, gel bands were washed three times with 25 mM NH_4_HCO_3_, 50% ethanol and then dehydrated with pure acetonitrile. Reduction and alkylation of proteins were performed by incubating gel samples in 10 mM DTT, 100 mM NH_4_HCO_3_ for 30 min at 56 °C and alkylation in 55 mM iodoacetamide, 100 mM NH_4_HCO_3 _for 45 min at RT. After wash in 25 mM NH_4_HCO_3_, 50% acetonitrile, gel samples were dehydrated with acetonitrile before being rehydrated with 40 μl of trypsin solution (400 ng in 50 mM NH4HCO3, 10% acetonitrile) for overnight digestion at 37 °C. Supernatants were transferred in a new tube and peptides were extracted twice with 50 μl of 50% acetonitrile, 0.1% TFA. Tryptic peptides were dried and resuspended in 10 μl of 50% acetonitrile, 0.1% TFA. Liquid Chromatography - Mass Spectrometry (LC-MS/MS) analysis were performed on an Ultimate 3000 Nano HPLC instrument (Thermo Fisher Scientific, Courtaboeuf, France) coupled to an Esquire HCTultra ion trap mass spectrometer (Bruker Daltonics, Marne-la-Vallée, France). The LC separation was performed on a PepMap100 column (0.075 mm ID, 3 μm particle size, 15 cm long; Thermo Fisher Scientific) at a flow rate of 200 nL/min, employing a linear gradient from 0% to 50% of H2O/acetonitrile/formic acid (5/95/0.1 v/v) for 35 min. A search for protein identity was carried out with MASCOT software (http://www.matrixscience.com) using the PlasmoDB protein database (http://plasmodb.org/plasmo/). Confident matches were defined by a MASCOT score above 28 (threshold score corresponding to statistical significance p < 0.05).

### CBPs transfectants in the HEK293 cell line

The CBP-DNAs were synthetized by the GeneArt Plasmid Services (Life Technologies) with the CX3CL1 signal peptide and codon optimization for human cells. They were cloned in pMA gene art vector between SacI and KpNI restriction sites. These constructs were digested with HindIII and BamHI and the HindIII/BamHI fragments were then cloned in pEYFP-NI (Clontech, Ozyme, France). CBP-pEYFP constructs were transfected into HEK293 using the cationic polymer transfection reagent jetPEI (Polyplus transfection, Illkirch, France). Stable transformants resistant to 1 mg/ml G418 (Life Technologies) were selected with Facs AriaII Cell Sorter (Becton-Dickinson, le Pont de Claix, France).

### Static adherence assay

CX3CL1 or CCL2 were adsorbed overnight at 4 °C on flat-bottom 96-well microtiter plates (Nunc A/S, Roskilde, Denmark) at the indicated concentrations (50 μl/well) diluted in 25 mM Tris pH 8 and 150 mM NaCl. Well surfaces were blocked for 2 h at room temperature (RT) with 1% nonfat milk in the same buffer. Enriched iRBCs were suspended at 5 × 10^6 ^ml-1 in HBSS supplemented with 1 mM CaCl_2_, 1 mM MgCl_2 _and 10 mM Hepes (pH 6.8). The CBPs-HEK293 transfectants to be tested were first labelled with 1 mM 5(6) carboxyfluorescein diacetate succinimidyl ester (Interchim, Montluçon, France). Before addition, enriched iRBCs and CBPs-HEK293 transfectants were treated or not for 15 min at RT with 500 nM CX3CL1 or CCL2, or with 0.5 μg/ml of the different Abs. Alternatively, the wells with the immobilized CX3CL1 chemokine were incubated with peptides CBP1-EC and CBP2-EC as well as CBP1-ECrd and CBP2-ECrd at 0.1 μM. The cells (1.5 × 10^6^ iRBCs or 10^4^ HEK293 cells per well) were then added and incubated 1 h at 37 °C. This cell number was adjusted to be sure they form a cell monolayer at the well bottom. Then this cell concentration was quantified by measurements of nine different fields per well by absorbance (415 nm) for iRBCs and by fluorescence (excitation 487 nm, emission 538 nm) for HEK293 cells (Flexstation 3, Molecular Devices, Sunnyvale, USA). To remove non-adherent cells, the wells were gently filled with PBS supplemented with 1 mM CaCl_2_ and 1 mM MgCl_2_ and the microplate was placed floating upside down for 1 h in a PBS/Ca/Mg solution as described as previously^51^. After this washing step, a new absorbance/fluorescence measurement was performed. Its ratio to the first one gave access to the number of adherent cells as a ratio to the number of total cells loaded in each well. The number of adherent cells per mm^2^ was then calculated (Figs 1A–D and 3B) knowing that the well surface is 0.32 mm^2^. In experiments evaluating inhibition of iRBC adherence by antibodies and peptides to normalize several experiments (Figs 2A, 6 and 7B), the number of adherent cells was expressed as % of the control, i.e. the number of adherent iRBC on coated CX3CL1 (50 pmoles/well).

### Flow adherence assay

The day prior the assay, CX3CL1-L929 transfectant and parental cells (10^4^ cells in 20 μl DMEM containing 10% FCS) were platted in IbiTreat −15 μ-SlideVI (Ibidi Biovalley, Marne La Vallée, France). The slides were examined by an inverted microscope (TE300, Nikon, Champigny, France) equipped with a phase contrast 10 ×  objective (Nikon, n.a. 0.25) and a cooled CCD camera (Sensicam, PCO, Kelheim, Germany). iRBC (parasitemia superior to 80%) were resuspended in flow buffer HBSS (GE Healthcare Europe, Velizy-Villacoublay, France) supplemented with 1 mM CaCl_2_, 1 mM MgCl_2_ (pH 6.8) at 5 × 106 mL-1 and incubated 15 min at 37 °C in the presence or not of 0.5 μM CX3CL1 or CCL2. A syringe pump (PHD 2000; Harvard Apparatus, Les Ulis, France) drove 0.5 ml of iRBC suspension through the IbiTreat slide at a wall shear stress of 0.27 dynes.cm^−2^. The 37 °C temperature was controlled all over the experiment. After a 10 min wash, images of fifteen 0.57 mm2 separate fields were recorded to count the adherent cells. The results were expressed as mean ± SD per mm^2^.

### Fluorescence imaging

To prevent nonspecific staining, the diluted rabbit sera were adsorbed on fresh normal human O+ red blood cells. In order to explore proteins expressed at the membrane surface, unfixed iRBCs were used in liquid phase. Pellets (5 μl) of cultured iRBC were incubated for 45 min at RT with 100 μl of serum diluted 5 times in PBS supplemented with 2% BSA and 100 μg/ml Hoechst reagent. After washing with PBS supplemented with 2% BSA, antibodies were detected with Alexafluor 488 conjugated goat anti-rabbit IgG (Life Technologies). Immunofluorescence staining was analyzed with a direct microscope (BX51, Olympus, Rungis, France). Fluorescence imaging of the HEK293 clones expressing the CBP candidates as EYFP chimera was performed on Lab-Tek II slides (Nunc, Dutscher) after fixation (PFA 4%, 30 min 4 °C) using an Olympus IX81-ZDC2 inverted microscope equipped for Total Internal Reflection fluorescence (TIRF). Digital images were captured on a cooled charge-coupled device (CCD) camera (ORCA-ER, Hamamatsu, Massy, France).

### Molecular interaction fluorescence assay

Solutions of 30 μl of 500 nM of each of the 5-TAMRA fluorescent peptides were prepared at 4 °C in the presence or not of 2 μM of CCL2 or CX3CL1 using the HBSS buffer supplemented with 1 mM CaCl_2_ and 1 mM MgCl_2_ in a 96-well white plate. After 20 minutes of incubation at RT the 5-TAMRA fluorescence was measured (excitation 520 nm, emission 580 nm) using the Flexstation 3 Microplate Reader.

### Statistical analysis

Data are expressed as mean and standard deviations of replicates as indicated in the legend of Figures. Analysis of statistical significance were done either by Student’s t-test or analysis of variance (ANOVA) followed by Tukey test. All statistical analysis were performed using Prism 5.2 (GraphPad Software, San Diego, USA). The levels of significance were indicated as following: *p ≤ 0.05; **p ≤ 0.005; ***p ≤ 0.0005; not significant (ns): p > 0.05.

## Additional Information

**How to cite this article**: Hermand, P. *et al. Plasmodium falciparum* proteins involved in cytoadherence of infected erythrocytes to chemokine CX3CL1. *Sci. Rep.* **6**, 33786; doi: 10.1038/srep33786 (2016).

## Supplementary Material

Supplementary Information

## Figures and Tables

**Table 1:** Characteristics of the five parasite proteins selected as potential CBPs.

Name in PlasmoDB	Gene Family	Acronym	Chromosome number	mRNA (bp)	Intron number	Signal peptide	TM number	Sequence (AA)	MW (Da) with signal peptide	MW (Da) without signal peptide
PF3D7_0113900 GEXP10	hyp8	CBP1	1	732	1	Yes	2	243	28234	25320
PF3D7_1301700 GEXP07	hyp8	CBP2	13	738	1	Yes	2	245	28255	25328
PF3D7_0501000			5	783	1	No	1	260	30716	
PF3D7_1478100	hyp13		14	744	1	Yes	2	247	28657	25553
PF3D7_1102600 GEXP14			11	816	1	No	3	271	32328	

Among the hits with significant scores identified by proteomics, five genes coding for proteins with a 20–35 kDa molecular mass and containing a Pexel motif were selected. Their characteristics are displayed according to the PlasmoDB (http://plasmodb.org/plasmo/).
