# Supplementary material for: Plasmodium falciparum proteins involved in cytoadherence of infected erythrocytes to chemokine CX3CL1
Source: Sci Rep. 2016 Sep 22;6:33786. doi: 10.1038/srep33786 (PMC5031962; doi:10.1038/srep33786)
Supplement: Supplementary Information [file srep33786-s1.pdf]

Plasmodium falciparum proteins involved in cytoadherence of infected erythrocytes to chemokine CX3CL1

Patricia Hermand[1], Liliane Cicéron[2], Cédric Pionneau[3], Catherine Vaquero[1], Christophe Combadière[1] and Philippe Deterre[1*]

[1]Sorbonne Universités, UPMC Univ Paris 06, Inserm, Centre d'Immunologie et des Maladies Infectieuses (Cimi-Paris), UMR 1135, ERL CNRS 8255, 91 boulevard de l'Hôpital F-75013, Paris, France

[2] Sorbonne Universités, UPMC Univ Paris 06, Inserm, Centre d'Immunologie et des Maladies Infectieuses (Cimi-Paris), UMR 1135, 91 boulevard de l'Hôpital F-75013, Paris, France

[3]Sorbonne Universités, UPMC Univ Paris 06, Plateforme Post-génomique de la Pitié-Salpêtrière (P3S), UMS 2 Omique, Inserm US029, 91 boulevard de l'Hôpital F-75013 Paris France

[*]Corresponding author: philippe.deterre@upmc.fr

Supplementary Information

SUPPLEMENTARY METHOD

ELISA against CBP-EC peptides

Flat-bottom 96-well microtiter plates (Nunc) were coated over night at 4°C with 5 and 25 ng/well peptides (CBP1-EC, CBP1-ECrd, CBP2-EC, CBP2-ECrd) in 50 mM sodium carbonate buffer (pH 9,6). Nonspecific binding sites were blocked for 1 hour at 37°C with PBS containing 2 % non-fat milk. The human sera were diluted at 1 :10, 1 :20 and 1 :40 in PBS with 2% non-fat milk and incubated for 1 hour at 37°C. Then the wells were washed four times in PBS with 0.05% Tween 20. The Horseradish peroxidase (HRP)-conjugated secondary antibody (Sigma) was diluted at 1:2000 dilution in PBS with 2% non-fat milk and incubated for 1 hour at 37°C, and washed with the same protocol. HRP activity was visualized using TMB Elisa substrate solution (eBioscience). Activity was blocked by adding 1 M sulfuric acid and optical density (OD) was determined at 450 nm (Flexstation reader). We used plasma from 19 patients living in or recent migrants from (less than 2 years) high malaria endemic countries (West and Center Africa). All these plasma were found to be negative in an ELISA assay against the CBP1-EC and CBP2-EC peptides. As control, the anti-CBPs antibodies displayed positive responses when used at dilution 1:50 over 5 ng/well of CBP1-EC or CBP2-EC. These positive responses were specific since no response was observed using CBP1-ECrd or CBP2-ECrd in the same conditions.

SUPPLEMENTARY FIGURE S1.

Adherence of 3D7-iRBC subjected to panning on the CX3CL1 chemokine.

A) Expression of CX3CL1 on parental L929 cell line (grey histogram) and the CX3CL1 positive L929 clone (empty histogram) was tested with phycoerythrin-labelled murine anti-CX3CL1 mAb (mAb clone 51637, R&D Systems, Lille, France) and analyzed by flow cytometry with a FACScalibur (Becton Dickinson, Le Pont-de-Claix, France). B) Static adherence in 96-well plates treated (grey bars) or not (empty bars) with 25 pmoles of CX3CL1 of 3D7-iRBC submitted (panned) or not (unpanned) to four rounds of selection over L929 cell line clone expressing CX3CL1. Enriched mature iRBC were incubated for 1h at 37°C on L929-CX3CL1 cells with 80% confluence in cytoadherence medium (RPMI 1640 pH 6,8). After five washes to remove unbound iRBC, RBC (5% hematocrit) in complete medium was added in the flask. 24h later, the younger ring stage parasites were transferred in another flask and cultured as described previously. After four rounds of selection, the iRBC were tested in static adherence as described in Experimental procedures. The number of adherent RBC per mm^2 was expressed as mean values and standard deviations from three independent experiments. ** $p \leq 0.005$

SUPPLEMENTARY FIGURE S2.

Specific adherence of HEK293 clones expressing CBP1 and CBP2.

A and C). Static adherence of HEK293 clones expressing CBP1 (A) or CBP2 (C) in 96-well plates coated with various concentration of CX3CL1 (grey circles) or with 50 pmole per well of CCL2 (black circles). The number of adherent cells per mm^2 was expressed as mean values and standard deviations from four replicate wells. B and D) Static adherence in 96-well plates coated with 25 pmoles of CX3CL1 of HEK293 clones expressing CBP1 (B) or CBP2 (D) pretreated or not (control) with 500 nM of CX3CL1 or 500 nM of CCL2, or 0.5 µg/ml of anti-CX3CR1 or anti-DARC antibodies. The data are expressed as percent of the control, as mean values and standard deviations from three independent experiments. Analysis of variance (ANOVA) followed by post hoc analysis with Tukey test was performed to establish the levels of significance: *** $p \leq 0.0005$.

SUPPLEMENTARY FIGURE S3.

Analysis of *cbp1* and *cbp2* transcripts in iRBC from patients.

Total *P. falciparum* RNAs were extracted in Trizol (Life Technologies) from African patients' bloods treated with DNAseI (Qiagen) and purified using RNeasy microcolumns (Qiagen) according to manufacturer's protocols. Total RNA (1µg) was retro-transcribed using the SuperScript Vilo cDNA Synthesis Kit (Life Technologies). PCRs were carried out with 4 µl of each cDNA sample and conditions were 5 min at 94°C, followed by 30 cycles at 94°C for 30s, a specific annealing temperature 52°C for 30 s, 60°C for 1min and a final 10min step at 60°C. Primers for the amplification of CBP1 are forward (5'ATGAACATTTATATTAGGACCATTTTTTTTGC) and backward primers (5'TTATTGAAAATGTAATATTTGTCTTAATATTTTTTC) and for CBP2, forward (5'ATGTCCTTTTGTTACGTTAGAACAATTTCTATTGC) and backward (5'TTAAAAATTAGAAACTTGTTTAATGATTCTTTCAC). The PCR products were resolved on 1% agarose gel and visualized under UV light after staining with ethidium bromide. Sequencing the amplicons confirmed that they are *cbp1* and *cbp2* transcripts.

SUPPLEMENTARY FIGURE S4.

Flow cytometry of 3D7-iRBC using anti-CBP antibodies.

Intact mature 3D7-iRBC (80% parasitaemia) and uninfected RBC were stained using 10µg/ml anti-CBP1 and anti-CBP2 rabbit polyclonal antibodies plus secondary goat anti-rabbit Alexa-Fluor 488 antibody and analyzed with a FACS-Canto (Becton Dickinson).

SUPPLEMENTARY FIGURE S5.

Patient iRBC staining with anti-CBP1 and anti-CBP2.

Cameroonian patient's erythrocytes were washed three times in prewarmed (37°C) RPMI to remove buffy coat. The erythrocytes suspension was added to a cell culture flask in complete medium 24h at 37°C for maturation and then staining with antibodies as described in Experimental Procedures. iRBC were visualized by transmitted light, by staining using Hoechst (bleu) and anti-CBP1 or anti-CBP2 antibodies (green). Bar = 10 µm.

SUPPLEMENTARY FIGURE S6.

The antibodies raised against CBP1 and CBP2 stain the sexual forms of *P.falciparum* in 3D7-iRBC

The staining was performed as described in Methods section after cell fixation (PBS supplemented with 4% PFA, 0.025% glutaraldehyde). The gametocytes preparation was a generous gift of Agnès Zettor (Centre de Production et d'Infection des Anophèles, Institut Pasteur, Paris)

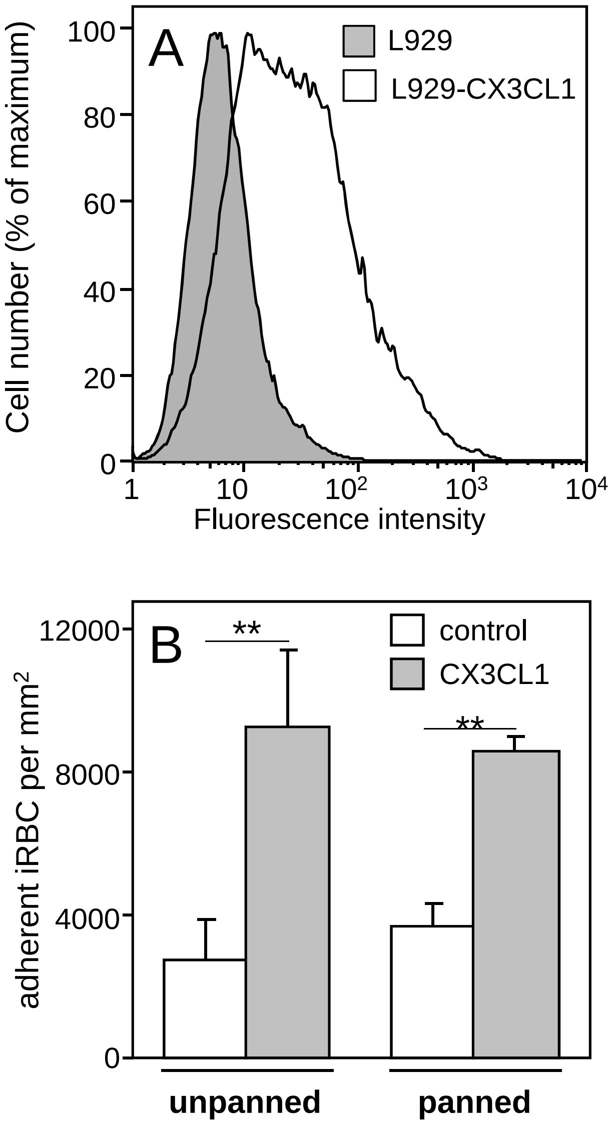

Hermand et al.
Supplementary Figure S1

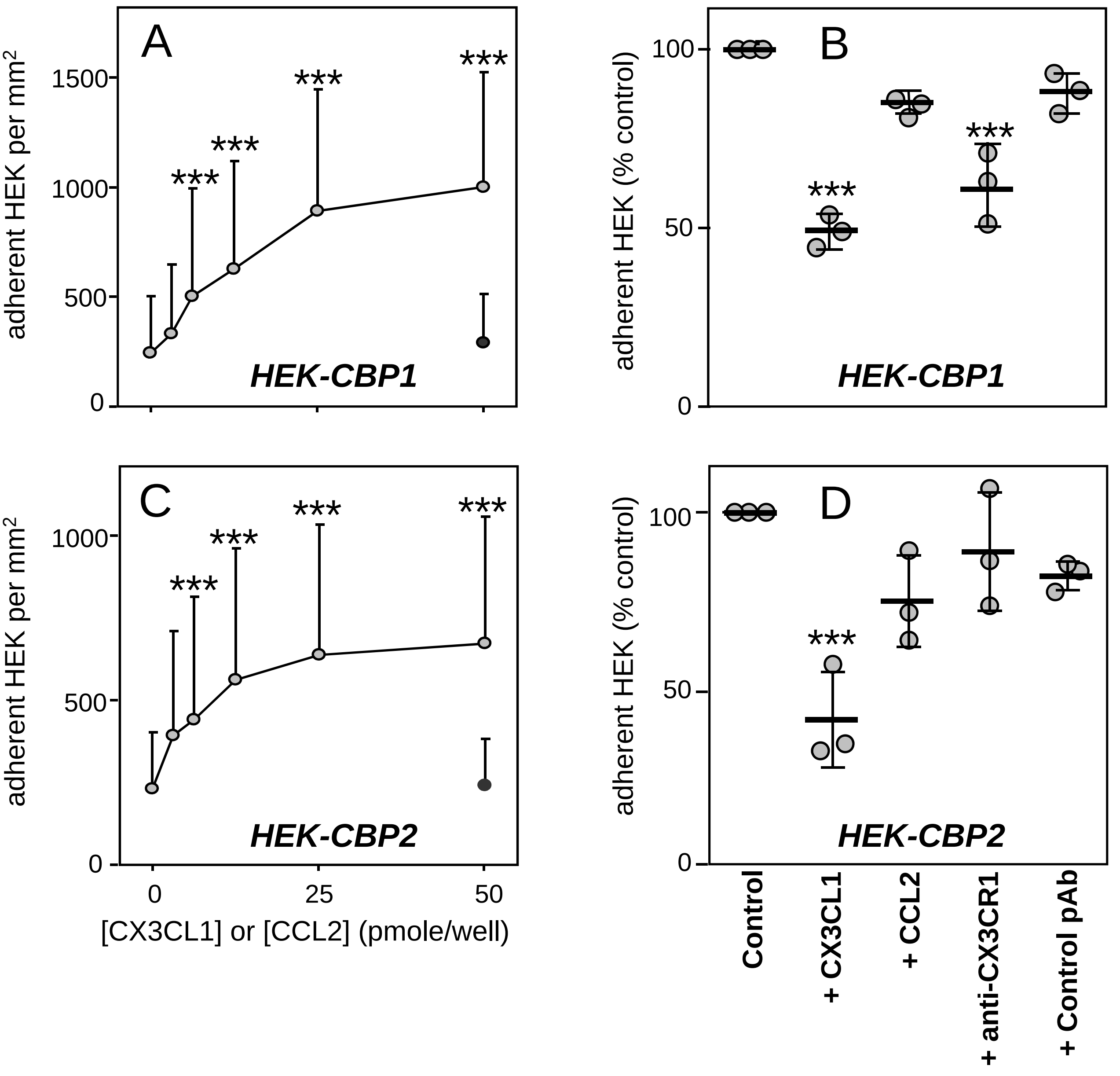

Hermand et al.
Supplementary Figure S2

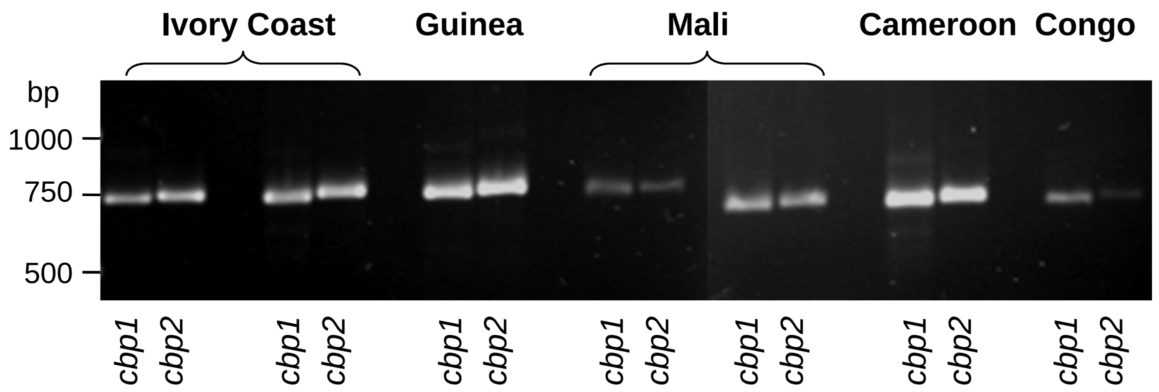

Hermand et al.
Supplementary Figure S3

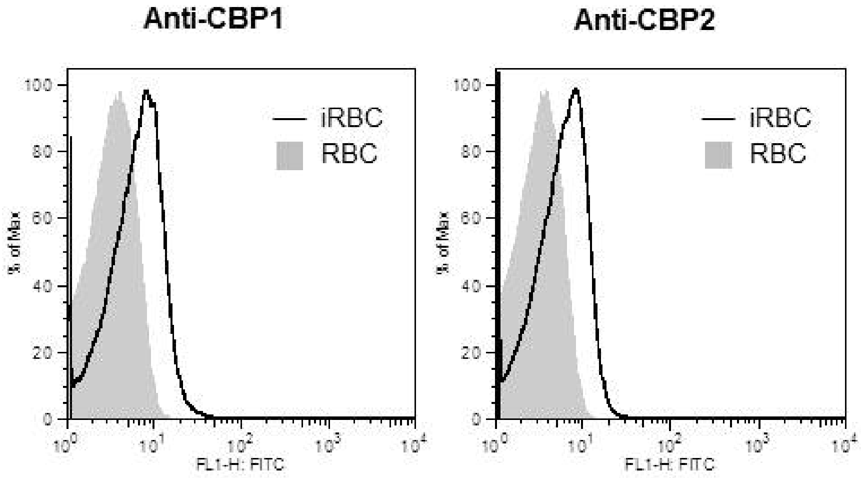

Hermand et al.
Supplementary Figure S4

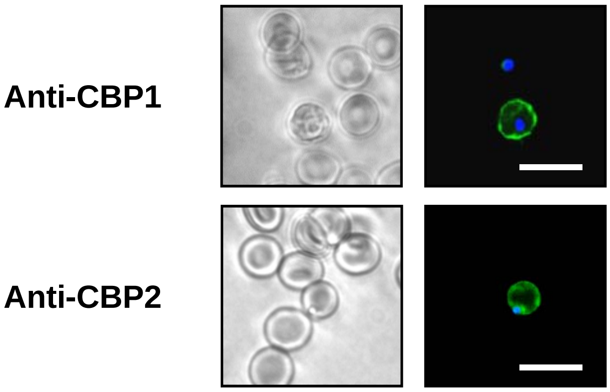

Hermand et al.
Supplementary Figure S5

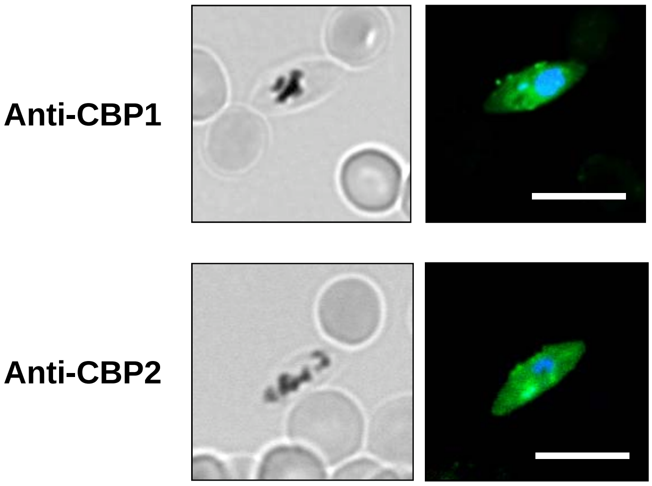

Hermand et al.
Supplementary Figure S6
